# Textual reading comprehension and naming in Alzheimer’s disease patients

**DOI:** 10.1590/S1980-57642009DN20200010

**Published:** 2008

**Authors:** Juciclara Rinaldi, Gabriela Sbardelloto, Christian Haag Kristensen, Maria Alice de Mattos Pimenta Parente

**Affiliations:** 1 MSc in Psychology, Universidade Federal do Rio Grande do Sul, Porto Alegre RS, Brasil.; 2 BSc in Psychology, Universidade Federal do Rio Grande do Sul, Porto Alegre RS, Brasil.; 3 PhD, Pontifícia Universidade Católica do Rio Grande do Sul.; 4 PhD, Universidade Federal do Rio Grande do Sul, Porto Alegre RS, Brasil.

**Keywords:** Alzheimer's, textual comprehension, lexical production, memory, language

## Abstract

Among linguistic-cognitive failures, the retelling of stories and lexical disorders occur from the onset of AD. Recent studies have discussed whether lexical failures in AD patients include naming actions.

**Objectives:**

The aims of this study were to verify naming and reformulation of action difficulties in AD patients and their relationship with the retelling of stories. Our main questions were: Are there two linguistic abilities impaired in the early stages of AD? Is there some correlation between the capacity of naming actions and the retelling of stories?

**Methods:**

We assessed 28 elderly participants: 17 with probable AD and 11 control subjects, with schooling ≥4 years. The textual reading comprehension was measured using four stories with descriptive and narrative textual structure. The lexical production was verified by 17 actions on video, assessed by the participants' first and second verbal emissions.

**Results:**

The results showed that the retelling of stories is a task that discriminates patients with AD from healthy individuals. The naming and reformulation of actions tasks did not show significant differences among the patients and their controls. A positive correlation was found between the difficulties in retelling stories and the reformulation of the naming of actions.

**Conclusions:**

These results confirm previous findings that show the preservation of naming actions in patients with AD, which involve familiar actions, and that the retelling of short stories is an instrument that discriminates patients with AD from healthy elders. Results also suggest that the difficulties in retelling are related a breakdown in reformulating information, perhaps stemming from mechanisms of decreased memory work.

## Abstract (pt)

Entre as falhas lingüísticas, o reconto de histórias e os problemas lexicais ocorrem desde o início da DA. Estudos recentes têm discutido se as falhas lexicais nos pacientes com DA incluem a nomeação de ações.

**Objetivos:**

Os objetivos deste estudo foram verificar as dificuldades de nomeação e reformulação de ações em pacientes com DA e sua relação com o reconto de histórias. As principais questões que nortearam esse estudo foram: As duas habilidades lingüísticas estão prejudicadas no início da DA? Há alguma correlação entre a capacidade de nomeação de ações e de reconto de histórias?

**Metodos:**

Foram avaliados 28 idosos: 17 com DAs prováveis e 11 sem demência, com escolaridade ≥4 anos. A compreensão de leitura textual foi avaliada através de quatro histórias com estrutura textual descritiva e narrativa. A nomeação foi verificada por 17 ações em vídeo, tendo primeira e segunda emissão para cada participante.

**Resultados:**

Os resultados mostraram que o reconto de histórias é uma tarefa que discrimina pacientes com DA de idosos normais; já as tarefas de nomeação e de reformulação de ações não apresentaram diferenças significativas entre os pacientes e seus controles. Foi encontrada uma correlação positiva entre as dificuldades de reconto de histórias e a reformulação da nomeação de ações.

**Conclusões:**

Esses resultados confirmam achados anteriores que mostram a preservação da nomeação de ações em pacientes com DA, nas ações familiares e que o reconto de histórias curtas é um instrumento que discrimina pacientes com DA de idosos normais. Também sugerem que as dificuldades de reconto estão relacionadas a falhas de reformulação de informações, talvez oriundas de mecanismos de memória de trabalho diminuída.

The cognitive functions of language and memory are compromised in the initial stages of Alzheimer’s disease (AD). Among the language tasks that are impaired in the initial stages of AD are the understanding of textual reading and lexical production. The difficulties in understanding textual reading in these patients are known to be attributed to cognitive flaws, such as those involving episodic memory, short term memory (working memory) and attention, found early in the disease. Reports from^1-4^ outline that the elderly without dementia remember more items of information than those with dementia. These studies show that the retelling of stories is an accurate procedure to discriminate the disease.^5^ On the other hand, lexical difficulties, verified through the naming of pictures, have been said to be due to flaws in the lexical access or to semantic memory flaws. As the studies about linguistics disturbances in patients with AD have focused on these two activities separately, little is known about how these lexical difficulties interfere in the retelling of stories.

The understanding of contextual reading demands a variety of abilities from the language beholder, such as codification of text information, ability to retain the ideas read, search of textual coherence and narrative reproduction. Different models have been proposed in order to understand how people comprehend a text, taking into consideration the constructive process the reader performs during textual comprehension.^6^ Labov and Waletzky^7^ present a structural model for narratives. These are composed of five parts called macrocategories. The narrative starts with the *orientation and situation* macro category, informing the reader where, when, who and the situation (what); moving on to *complication*, that presents a series of events. Subsequently, the *complication* macrocategory introduces an event (following an action) and a problem, which will be solved or ended by the *resolution* macrocategory. Between complication and resolution lies the evaluation macrocategory that compares narrative units within the same structure. The resolution macrocategory ends the actions, while some narratives have one more macrocategory, the *conclusion* of the story offering a summary of what happened.

No studies were found verifying whether one of these macrocategories hampers comprehension of stories for patients with AD. Nevertheless, studies on patients with AD have shown that the kind of textual structure may facilitate comprehension, as evidenced in the comparative study between patients with AD and elderly patients without dementia, in which the participants have to retell 4 stories, 2 narrative and 2 descriptive.^3^ Within the perspective of the model,^7^ the descriptions are texts with a predominance of the first macrostructure, the situation/orientation component. In a study by Gély-Nargeot, Ska and Touchon,^3^ not only the patients with AD but also those without dementia presented a better performance when the structure was of a narrative. These authors also verified that more detailed versions, that is, more redundant, improve the comprehension of patients with AD. On the other hand, the results have shown that remembering, not only the main idea but also the details, decreases with the severity of the dementia, although in spite of this overall worsening, the main ideas are retained for longer than the details. The worsening of dementia, thus, besides impacting the story recall in patients with dementia, led them to remembering the main idea of the text over and above the details.^8-9^

Two models have proposed that text comprehension is a complex activity, with the participation of cognitive functions. Kintsch and van Dijk^10^ focused on the participation of short term memory (working memory) and long term memory (episodic memory) for textual comprehension. Due to the limited capacity of the short term memory, it is important to select the macrostructures of the text, that is, identify the main idea and discard the microstructures (details).^11-12^ Comprehension is performed in cycles, sent to the episodic memory, to build a base text. On the other hand,^13^ the relationship between cause and consequence of the events are emphasized in the text. According to this model, the comprehension of the narrative is stored in the long term memory (semantic memory) as a network, and the nodes of this net (sentences in the text) are interconnected by the causative relations.^14^ Under this model, the possibility of creating inferences, based on the four criteria of casual relation is important. Furthermore, the same model assumes that comprehension is based on the possibility of solving problems, and is not concerned about memory.

Retelling a story is not reproducing it word by word. Memory mechanisms retain the plot or the script and a variety of significant units of the story. The latter are called propositions. Each proposition is formed by an argument and a predicate. The argument corresponds to an action (verb) and the predicate may be who performs the action, suffers its consequences, etc. The nucleus of the narrative information is, thus, based on the actions and it is possible that the retelling of stories is related to the patient’s capacity of naming these actions.

Studies on the lexical difficulties of patients with AD have shown dissociation in the flaws in naming actions and objects. From the neuroanatomic point of view, there is evidence that various areas are responsible for the processing of these two word classes. While common and proper nouns are processed by posterior and temporal regions, verbs are represented in frontal circuits.^15-16^ Some neuroimaging studies (PET and RMf) however, show mixed representations that do not support this antero-posterior dissociation.^17^ Tyler, Russell, Fadili Cappa^18^ also found an extensive area of semantic processing that extends from the inferior frontal cortex up to the temporal lobe of the same side, with no grammatical class differentiation of words.

Comparing the task of naming of actions and objects performed by patients with AD versus patients with front temporal dementia (FTD),^19^ has shown that both groups were worse in naming compared to their controls in both classes, while patients with FTD were significantly better at naming objects compared to patients with AD. Overall, maintaining the ability to name actions with difficulties in naming objects is explained by the anatomical organization, since in the early stages of AD the posterior areas responsible for naming objects are more affected than the anterior ones, responsible for naming actions.

Another study group found the opposite pattern where verbs were more affected than nouns.^20-23^ This pattern is explained through the semantic characteristics of the verbs. Verbs are more complex, less imaginable and more abstract.

This work intends to fill a gap in the literature concerning the linguistic difficulties of patients with AD, studying the same group of participants for the capacity of retelling a story, naming verbs and retelling with naming production (reformulation task). Based on previous studies, patients with AD are expected to perform worse on both tasks than their controls. Given the action is an extremely important feature to the information units of a narrative, one expects to find a correlation between the retelling tasks and naming of actions.

## Methods

Twenty-eight aged native speakers of Brazilian-Portuguese participated in this study (8 males and 20 females). Patients fulfilled the DSM-IV criteria for dementia^24^ and the NINCDS-ADRDA for probable AD,^25^ as well as the Clinical Dementia Rating scale – CDR.^26^ The Mini Mental State Examination (MMSE) was also applied,^27-28^ and patients with scores between 24 and 27 were included in the group of mild AD, whereas patients with scores between 12 and 24 were included in the moderate group. Cognitively normal individuals were selected from within the community and only those with scores greater than 27 on the MMSE were selected. All subjects were submitted to the GDS-15^29,30^ scale, and those with scores lower than 10 were included in this study. Other criteria for inclusion were: schooling greater than four years, lack of history of any psychiatric or neurological disease, and visual or hearing impairment without correction. Table 1 shows the demographic characteristics of subjects in the three groups.

**Table 1:** Mean±SD demographic characteristics of Alzheimer's disease patients and normal elderly subjects.

Variables	Normal elderly (n=11)	Mild Alzheimer's patients (n=8)	Moderate Alzheimer's patients (n=9)	All Alzheimer' spatients (n=17)
Age (years)	78.27±6.51	76.75±6.76	80.13±4.49	78.44±5.80
Education (years)	8.00±3.64	7.88±3.64	7.00±1.85	7.44±2.83
MMSE (maximum=30)	27.55±1.44	21.63±1.19	15.67±1.32	18.47±3.30
Gender (male/female)	3/8	4/4	1/8	5/12

MMSE: Mini-Mental State Examination.

### Materials and procedures

Two experimental tasks were selected for this study: a reading comprehension task and a verb naming task.

### Reading comprehension task

**Figure 1:**
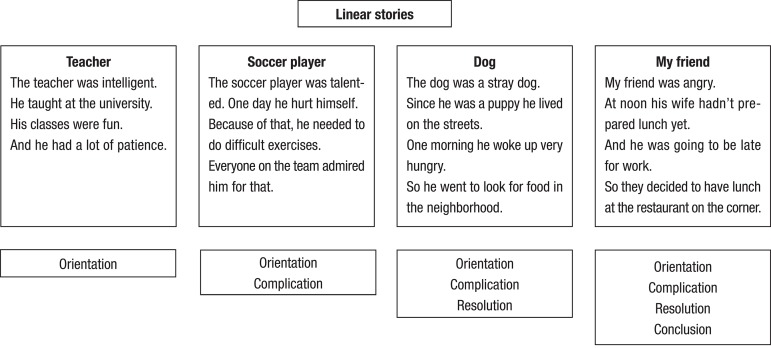
Stories and macrocategories used in textual reading comprehension.

Four stories, each composed by four sentences were presented visually to the participants in a presentation on a computer. Four macro-categories were controlled in the construction of the stories. The first story had only the situation/orientation category, and thus was similar to a description. The second story had situation/orientation and complication; the third situation/orientation, complication and resolution and the fourth, all four categories^7^ (Figure 1). The computer presentation was controlled by the E-Prime Program. A dark blue notebook screen (17”) showed the first sentence in the upper part in white letters. In the lower part, the following sentence was written in yellow. The participant was instructed to read the sentence and when finished to hit a key on the computer. The following sentence appeared in the upper screen. After reading the four sentences the participants were asked to retell the story. The patient’s recall was recorded and later transcribed. For each story, participants received a score of percentage of the number of propositions recalled that were similar to those presented in the original story.

### Verb naming and reformulation tasks

A verb naming task was elicited by the presentation of 17 different short videos. In each film, the same sound indicated the beginning of the movie. A red curtain opened and a masquerade woman walked towards a table where several objects were displayed. She selected a different object each time and performed an action of destruction or separation of the object. For example, a balloon was burst with her hand or a doll was undressed. Videos were presented individually in Windows Media Player format, and the length of each film varied from 42sec to 1min 13sec.^31^ Films were shown to participants in a random order (Table 2). At the end of the action, the examiner asked participants “What has the woman done?”

**Table 2:** Number of film, verbs, objects and actions of the naming and reformulation of actions tasks.

Action
To peel a slice of a tree trunk
To blow up a balloon
To peel a banana
To peel a carrot
To partially rip the sleeve of a shirt
To tear a newspaper in half
To break a glass with a hammer
To dismantle a small Lego castle
Partially peel an orange
To cut a loaf of bread in half with a bread knife
To screw up a sheet of paper
To saw a board in half
To undress a doll
To squash a tomato with a slap
To break a loaf of bread in half with the hands
To crumble a toasted loaf of bread
To chop a bunch of parsley with a kitchen knife

The reformulation task followed the naming task. After naming, the examiner asked “Tell me what the woman has done using another word.” All answers were tape-recorded and subsequently written down. Each response was classified according to: 1 - *Conventional response:* a response with a verb where the verb was considered, from a semantic and pragmatic point of view, to belong to the semantic domain of the action shown in the film. 2 - *Non-conventional responses:* were those verbs that showed a semantic relationship to the action in the film, but their use was not accepted as adequate for the linguistic community to denote the action depicted in the film. Three independent raters scored all verbs produced by the participants. The final score was obtained by intra-rater agreement. The study was approved by the Ethics Committee for Research. Participants or their guardians consented to participate before enrolling on the study.

### Statistical analyses

Descriptive statistics (mean, SD, and relative frequency) were calculated for demographic data, MMSE, verb naming and data and text scores (score of each story, mean score of the four stories and the best remembered story).The Kruskall-Wallis test was used for comparison of the three groups. Spearman’s rho correlation coefficients were calculated for correlations between MMSE, text scores and verb naming and reformulation data. The statistical analysis was carried out by the Statistical Package for the Social Sciences for Windows (SPSS 11).

## Results

### Are there group differences in naming and reformulation tasks of actions?

Descriptive statistics of naming action and reformulation tasks of AD patients and their controls are shown in Table 3. On naming task and reformulation tasks, no statistically significant effects were observed for comparisons between AD and their controls (naming: Kruskall-Wallis test, chi-square=0.974; p=0.324; reformulation: Kruskall-Wallis test, chi-square=0.688; p=0.407). Also, no significant differences were found between mild and moderate AD patients in both naming and reformulation tasks (naming: Kruskall-Wallis test, chi-square=.096; p=0.757; reformulation: Kruskall-Wallis test, chi-square=1.033; p=0.309)

**Table 3:** Mean ±SD in naming actions and in reformulation task scores of Alzheimer's disease (AD) patients and normal elderly subjects.

	Naming actions	Reformulation tasks
Effect	Mean±SE	Mean±SE
Mild AD patients	15.50±0 .92	10.87±4.45
Moderate AD patients	15.22±1.09	8.11±5.44
All AD patients	15.35±1.00	9.41±5.05
Controls	15.64±1.20	11.36±2.54

### Are there group differences in text recall?

To study text recall, we obtained six different scores: one score that corresponded to the mean recall of the four texts; one score of the story best recalled by each subject, and four individual scores that express the recall of each text. Table 4 shows their means and standard deviations. Performance on text recall differed significantly between AD patients and their controls in all six measures (mean recall score Kruskall-Wallis test, chi-square=16.942; p<.001; best recalled story Kruskall-Wallis test, chi-square=15.918; p<.001; descriptive story Kruskall-Wallis test, chi-square=12.590; p<.001; story ended in the complication Kruskall-Wallis test, chi-square=11.194; p=.001; the story ended in the resolution Kruskall-Wallis test, chi-square=12.012; p=.001; and story with all narrative elements Kruskall-Wallis test, chi-square=15.918; p<.001). Controls’ scores were significantly better than mild AD in all text measures (mean recall score Kruskall-Wallis test, chi-square=14.142; p<.001; best recalled story Kruskall-Wallis test, chi-square=14.392; p<.001; descriptive story Kruskall-Wallis test, chi-square=12.017; p<.001; story ended in the complication Kruskall-Wallis test, chi-square=11.829; p=.001; the story ended in the resolution Kruskall-Wallis test, chi-square=12.674; p=.001; and story with all narrative elements Kruskall-Wallis test, chi-square=13.743; p<.001). Comparison between Mild and Moderate AD patients showed significant differences in the mean recall score (Kruskall-Wallis test, chi-square=6.750; p=.009), in the best recalled story (Kruskall-Wallis test, chi-square=5.572; p=.18) in the descriptive story (Kruskall-Wallis test, chi-square=4.580; p=.32), in the story with all narrative elements (Kruskall-Wallis test, chi-square=4.793; p=.29), and in the story ended in the resolution (Kruskall-Wallis test, chi-square=4.464; p=.035). No differences were found in the recall of the story ended in the complication (Kruskall-Wallis test, chi-square=1.494; p=.222).

**Table 4:** Mean±SD in all text recall scores of Alzheimer's disease (AD) patients and normal elderly subjects.

Scores	Mild AD Mean±SE	Moderate AD Mean±SE	All AD Mean±SE	Controls Mean±SE
Mean (four stories)	54.33±22.04	26.72±17.05	39.71±23.66	81.22± 8.34
Best recalled text	66.49±23.09	43.04±21.60	54.07±24.74	92.57± 8.77
Descriptive story	60.71±22.59	32.14±26.17	46.42±27.84	84.41±11.87
Story ended in complication	54.17±24.80	36.46±22.24	45.31±24.52	81.05±12.96
Story ended in resolution	53.41±26.86	23.86±21.15	38.63±27.87	81.81±13.48
Story with all narrative macrostructures	49.04±26.31	21.37±20.27	34.38±26.66	77.62±12.14

### Does MMSE correlate with naming verbs and text recall measures?

Significant correlations were found between MMSE and all six text recall measures (MMSE and mean text recall score, Spearman rho=.822; MMSE and descriptive text recall score, Spearman rho=0.759; p<0.001; p<0.001; MMSE and text recall score of the story ended in complication, Spearman rho=0.728; p <0.001; MMSE and text recall score of the story ended in resolution, Spearman rho=0.751; p<0.001; MMSE and text recall score of the story with all narrative macrostructures, Spearman rho=0.753; p< 0.001). No correlation was found between MMSE and naming or reformulation tasks (naming, Spearman rho=214 p=0.274; reformulation, Spearman rho=0.363 p=0 .278).

### Do text measures correlate with naming action score measures?

As shown in Table 5, no correlations were found among naming action and all text recall scores, but significant correlation was found between reformulation of naming action and the best story recalled (Spearman rho=0.953; p<0.001), the descriptive story (Spearman rho=0.480; p=0.011) and the story ended in complication (Spearman rho=0.620; p= 0.001). No correlation was found between naming action and reformulation task (Spearman rho=0.620; p=0.001).

**Table 5:** Spearman correlations between naming action, reformulation task and text retelling scores.

		Naming	Reformulation	Mean text retelling	Descriptive story	Story ended in complication	Story ended in resolution	Story with 4 macro categories
Reformulation	rho p n	0.328 0.089 28						
Mean text retell	rho p n	0.127 0.519 28	0.053 28					
Descriptive story	rho p n	0.000 1.000 27	0.480(*) 0.011 27	0.918(**) 0.000 27				
Story ended in complication	rho p n	0.314 0.111 27	0.620(**) 0.001 27	0.897(**) 0.000 27	0.820(**) 0.000 27			
Story ended in resolution	rho p n	0.047 0.817 27	0.076 0.708 27	0.914(**) 0.000 27	0.738(**) 0.000 26	0.668(**) 0.000 26		
Story with macroestructures	rho p n	0.123 0.532 28	0.170 0.386 28	0.939(**) 0.000 28	0.783(**) 0.000 27	0.788(**) 0.000 27	0.853(**) 0.000 27	
Story best retold	rho p n	0.088 0.655 28	0.457(*) 0.015 28	0.953(**) 0.000 28	0.931(**) 0.000 27	0.908(**) 0.000 27	0.814(**) 0.000 27	0.843(**) 0.000 28

* Correlation is significant at the 0.05 level (2-tailed);

** Correlation is significant at the 0.01 level (2-tailed).

## Discussion

Our results showed that the retelling of stories is a task that discriminates patients with AD from healthy elders. In all the measurements obtained, the AD patients remembered significantly fewer propositions than their controls. This difference was found even among patients with mild AD and healthy elders. Among the elders with moderate AD and those with mild AD, only one story did not yield a significant difference. The results also show that even in very short stories, cognitive impairment measured by MMSE, is associated with the difficulty of common retelling in the elderly. Therefore, further studies involving a greater population of patients with AD could describe different levels of abilities according to mental decline, improving the communicative diagnosis of these patients.

As this text is a set of inter-related meanings, some results can evidence a degree of semantic influence. Our work did not evidence a major preservation of the narrative structure over the descriptive. Perhaps due to the control of the size of the sentences and the fact that the descriptive text was short and having the qualities of a teacher that could be summarized in one topic only, “the teacher was good”. The information became redundant, making memorization easy. On the other hand, factors related to the familiarity of the story could explain the good performance of the moderate AD patients compared to the mild AD, in the story that ended in complication. This story was about a domestic dog and its general meaning was frequently retold by the patients with moderate AD.

The naming of actions and their reformulation did not present any difficulties for our patients with AD. These two tasks did not inter relate with the scores on the MMSE. In relation to the correlation between the tasks of naming and textual retelling, only the reformulation task showed a positive correlation with the best told story by the participants. These results indicate the preservation of the naming of actions in patients with AD, at least for fairly familiar actions and when a wide aspect of conventional answers is considered (for example, for the action of sawing wood, besides the verb to saw, the verb to cut and to divide were also considered conventional). Is it possible that patients with AD use more generic verbs as a strategy to compensate for the semantic impairment?^21^

The correlation between the reformulation task and three scores of story retelling indicates that one of the processes involved in the difficulty in retelling for patients with AD is the process of reformulating textual information. Upon retelling, the individual looks for words associated to the meanings of the story and then reformulates it seeking to maintain the same meaning. This process likely involves mechanisms of working memory, which is very impaired in the early stages of AD.

This work has shown that the retelling of stories, even short ones, is compromised in patients with mild and moderate AD. The same does not hold however with tasks of naming and reformulation of actions. On the other hand, retelling of texts linked to the reformulation task, indicating that among the difficulties these patients encounter in retelling texts, flaws occur in reformulating the information of a text which is heard or read.

From a clinical point of view, the retelling of short stories seems to be an instrument able to discriminate patients with AD from healthy elders. Future studies should establish performance levels according to the severity of the disease, enriching the diagnosis and body of knowledge on the communicative capacity of this patient group.
